# What does the Malawi Demographic and Health Survey say about the country’s first Health Sector Strategic Plan?

**DOI:** 10.7189/jogh.09.010314

**Published:** 2019-06

**Authors:** Paul Kawale, Claudia Pagliari, Liz Grant

**Affiliations:** 1 Nkhoma Hospital, Nkhoma, Malawi; 2 Usher Institute for Population Health Sciences and Informatics, University of Edinburgh, Edinburgh, UK

Providing access to reliable and effective health services to improve health in low- and middle-income countries (LMIC) is a key objective of global development strategies, embodied in the United Nations Millennium Development Goals and Sustainable Development Goals. Underpinning these high-level goals is the concept of health system strengthening, a complex construct embedding theories and approaches to improving the public health sector through six “building blocks”. These include service redesign approaches to improve efficiencies in the care delivery system, improving governance to reduce waste and fraud, enabling access to medicines to support health equity, using health information systems to inform the monitoring and management of health care, and building human and financial resource capacities.

While most LMICs have embraced the goals of health system strengthening, they vary widely in the extent to which these have translated into strategies, actions and evidence. This article describes the key health system strengthening approaches employed by the Malawian government and international agencies working with them, as articulated in the nation’s Health Sector Strategic Plan (HSSP, 2011-2016), and seeks to understand its implementation through examining open-access reports, publications and articles. It also seeks to report outcomes of these interventions through comparing the country’s Demographic and Health Surveys (DHS) conducted in 2011 and 2016.

## HISTORY OF MALAWI’S HEALTH SYSTEM

Formalised in 1994 with the change to multi-party democracy, Malawi’s Constitution safeguards its citizens’ health as its 13th principle of national policy, set in the goal “to provide adequate health care, commensurate with the health needs of Malawian society and international standards of health care” in Chapter III Clause 13(c). In pursuit of this goal, in 1999 the Ministry of Health produced its strategic vision to strengthen Malawi’s health systems by the year 2020. This strategy defined the Essential Health Package (EHP), Malawi’s version of Universal Healthcare Coverage (UHC), a quantifiable, costed package of health services to be accessible by every citizen free of charge within 5 km. It also introduced the Sector Wide Approach (SWAp), a consortium of health sector stakeholders with a secretariat at the Ministry of Health. At the same time, Malawi embraced its “Vision 2020”, the country’s development vision for the year 2020, which acknowledged improvement of the availability, accessibility and quality of health services as one of the country’s social sector strategic challenges.

From 2004, Malawi’s health sector began to be guided by an annual Programme of Work (PoW) to deliver the EHP. Through the SWAp, government and its domestic and external stakeholders developed, implemented and monitored the annual PoW. In 2011, with key lessons from the evaluation of the PoW and SWAp, the Malawi Health Sector Strategic Plan (HSSP) succeeded the PoW to guide the health sector between 2011 and 2016.

In 2016, the Malawi Demographic and Health Survey (MDHS) [1] reported changes in the country’s socio-economic and health indicators following the country’s implementation of its second Malawi Growth and Development Strategy (MGDS II) and the Ministry of Health’s first HSSP, both covering the period 2011 – 2016. These are portrayed below, and compared to the situation in 2011 when these health systems strengthening strategies began, as depicted in the 2011 MDHS [2]. In view of their priority in the then-Millennium Development Goals and the country’s HSSP, it is particularly important to consider the contribution of HSSP interventions on access to, quality and outcomes of maternal, neonatal and child health services, prevention and treatment of HIV, and malaria control. These were approached from a health system strengthening perspective, particularly the service delivery building block. We also specifically provide our viewpoint of the health system’s human resources, information, governance/leadership and finance building blocks. Health commodities building block was a cross-cutting issue.

## TRACKING THE EFFECTS OF HSSP IN MALAWI

### Maternal and perinatal health

Malawi’s maternal mortality ratio improved from 675 deaths per 100 000 live births in 2011 [1] to 439 in 2016 [2]. However, documented neonatal mortality remains high in Malawi, and even reductions from 40 to 35 deaths per 1000 live births between 2011 [2] and 2016 [1] will fall short of the UN sustainability goal of 12 per 1000 live births by 2030 [3]. Distressingly, the mortality reported at Malawian health care facilities may under-represent deaths in the communities by as much as 20% [4]. Paradoxically, over the same period, perinatal death rates increased to 40 and 82 deaths per 1000 pregnancies in urban and educated women, respectively – the causes of which are not well understood, but nevertheless suggest a need for targeted prevention efforts among urban and educated women.

### Neonatal and child health

There were still few women in Malawi who brought their children to the clinic for check-up within two weeks of birth, reducing from 43.0% in 2011 to 42.4% in 2016 [1,2]. Despite HSSP interventions encouraging post-natal service uptake, there remains limited evidence of its improvement of maternal and neonatal outcomes [5], and the post-natal period has significant cultural beliefs in Malawi not easily addressed by the health system [6]. MDHS reported a decline in the rate of children dying before their fifth birthday in the five years, from 112 deaths to 63 per 1000 live births [1,2]. Interventions in Malawi that showed improvements in child health outcomes included the use of mobile phones to promote home based child care, women’s or care groups, and integration of child health into HIV and maternal health services.

**Figure:**
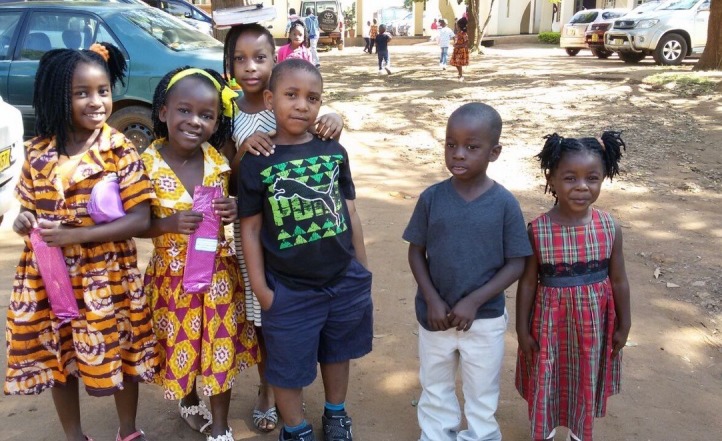
Photo: From the collection of Paul Kawale (used with permission).

### HIV

HIV prevalence in Malawi reduced from 10.6% to 8.8% between 2011 and 2016 [1,2]. This has been coupled with increased uptake of provider-initiated HIV testing services in Malawi [7] and successful HIV self-testing trials [8]. Over the same period, Malawi achieved a 68% reduction in new HIV infections among new-born babies, the highest change among global HIV priority countries [9]. However, although most women with HIV remain on treatment soon after childbirth [2], longer-term treatment retention in Malawi can go as low as 42%, particularly in the context of PMTCT Option B+, where a person found with HIV is enrolled into lifelong treatment, regardless of the state of their health, immune system or viral load. This low treatment retention has been attributed to logistical challenges, as well as socio-economic and cultural factors [10]. There is need for novel ways of retaining people in care to reach the global goals of 90% of people knowing their HIV status, 90% of people with HIV being on treatment, and 90% of those on treatment achieving viral suppression. It is also concerning that HIV prevalence remained high among widows, increasing from 31.8% to 50.1% prevalence between 2011 and 2016 [1,2].

### Malaria

Only 33.9% of the population used bed nets in 2016, slightly up from 29.0% in 2011 [1,2], reducing malaria by only 30% in Malawi [11]. However, prolonged widespread distribution of these nets in the country led to insecticide resistance [12], affecting the outcomes of the country’s indoor residual spraying (IRS) strategy for malaria vector control, such that the National Malaria Control Programme considered changing to a new type of insecticide for IRS [13]. Although it was described as the main strategy for malaria control in Malawi in the MDHS, only 2.2% and 4.9% of households had been sprayed in 2011 and 2016, respectively [1,2]. As malaria emerged as increasingly affecting school children, spraying boarding schools and their surrounding communities presents an effective strategy.

### Human resources for health

Investments aimed at strengthening human resources included sustained training and deployment of medical assistants to provide basic clinical care, particularly at rural facilities. Although started as a temporary measure until the country had enough medical doctors, they have become a core resource for Malawi’s health system. According to the WHO, a Malawian doctor serves 18 000 people as compared to an American or British citizen being served by approximately 3 doctors and every Cuban being served by at least 7 doctors. At community level, training and recruitment of additional community-based health surveillance assistants (HSAs) in Malawi significantly improved access to preventive and some curative health services. Although HSAs were reported to be motivated by their work, their peers and their communities, with 79.2% of community members expressing positive perceptions of their HSA, the HSAs have also reported being demotivated by their working conditions, with their managers conversely reporting negative opinions of the HSAs [14].

### Leadership/governance

Mid-level district health managers report that they perceive their supervisory role in health facilities in Malawi as a form of control and inspection, unlike those in neighbouring Tanzania, who regarded the role as support for improvement. In addition, even with intensive supervision of Malawian health workers, the improvements in basic knowledge and skills among primary health care workers have been very modest, with insignificant effects on clinical outcomes. However, data shows that in Malawi, a formal supervision process by any higher-level staff is associated with mid-level health workers’ improved job satisfaction and retention [15].

### Health information

In 2013, the Ministry of Health developed the Malawi Health Information Systems Strategic Plan and in 2014, the National Health Information System Policy and the eHealth Strategy were developed. Efforts to computerise the pharmacies so that drug logistics could be managed electronically, and implementation of an integrated electronic human resource management information system, were some of the government’s early digital health interventions. Pioneers of digital health systems in the country included Partners in Health (PIH), who implemented OpenMRS, which was modified by Baobab Health Trust to capture HIV, TB and later chronic diseases and primary care data. At district and national levels, the District Health Information System (DHIS) was implemented to capture aggregate data. Mobile technologies were also developing as health system strengthening tools in Malawi, including Chipatala Cha Pa Foni (CCPF) Project for strengthening maternal, neonatal and child health, Rapid SMS Project to strengthen surveillance of child nutrition, UNICEF's Programme Mwana strengthening early infant diagnosis of HIV and mother-infant postnatal visits, and John Snow Inc’s cStock to strengthen community supply of drugs.

### Finance

The Malawi Government has committed to finance the Christian Health Association of Malawi’s (CHAM) private non-profit facilities, enabling them to offer free maternal and child health services through service level agreements (SLAs). However, challenges in the distribution of funds from the Government has compromised CHAM’s potential to provide universal health care to the most vulnerable in Malawi. CHAM’s limited infrastructure in some clinics, and minimal human resources at some others, have also detrimentally impacted its capacity to deliver [16]. Nevertheless, improved facility usage has been reported resulting from SLA, such as 15% and 11% increases in antenatal visits and facility deliveries, respectively. We do not understand SLAs as a replacement to health system financing, and we do not believe Malawi’s Ministry of Health perceives it as such. It is complementary to the country’s existing health system financing mechanisms, aimed at expanding coverage of the EHP.

## LIMITATIONS

This viewpoint presents statistics from the DHS, which only presents one source of evaluation recommended in the HSSP. Although we tried to triangulate these data with published literature from the same period, this viewpoint needs to be received in the context of other assessments of the country’s health sector, such as the MDG country reports. It is beyond the scope of this brief viewpoint to evaluate each strategic goal of the HSSP (section 8.2). Readers should also be aware of the challenges involved in evaluating change in complex and dynamic environments, and there is need to further study the systemic barriers to health system strengthening, such as governance, using established indicators. This calls for qualitative research to understand how and why things may be changing, or are paradoxical, and/or to understand reporting biases. For example, the introduction of better community surveillance systems may lead to more reporting of health events or mortality, which might disguise actual improvements in health care, or external incentives may influence government’s reporting.

## CONCLUSION

Malawi’s population is expected to increase to 26 million by 2030. Its health system remains weak, partly due to sparse resources. Yet, despite these weaknesses, there have been significant achievements in reducing disease burden, achievements which many high-income countries have not been able to demonstrate in their identified areas of need. While low baseline capacity and performance in LMIC settings like Malawi go some way towards explaining the potentially greater impacts of the HSSP, the data nevertheless provides reassurance that development investments are paying dividends for the populations they are intended to benefit. Difficulties with the attribution of effects to interventions in real-world settings nevertheless represents a challenge for evidence-based policies, and much of the data reported here is merely statistical. However, the convergence of so many upward indicators does suggest positive impacts. With increased international research focused on Malawi’s health system and efforts to improve national research capacity, further insights and evidence are likely to emerge in the coming years. Despite these improvements, significant challenges remain in improving the population’s access to equitable, effective, efficient and affordable health care, and paradoxical indictors, such as reduced postnatal care uptake and perinatal deaths in urban and educated women, require further exploration and attention. With only one year to go to meet the goals set under Malawi’s Vision 2020 strategy, maintaining the push toward health system strengthening and future sustainability is essential, but the balance of indicators outlined here suggests that the effort is worth it.
